# Information-Theoretic ESG Index Direction Forecasting: A Complexity-Aware Framework

**DOI:** 10.3390/e27111164

**Published:** 2025-11-17

**Authors:** Kadriye Nurdanay Öztürk, Öyküm Esra Yiğit

**Affiliations:** 1 PhD Program in Statistics, Department of Statistics, Graduate School of Science and Engineering, Yildiz Technical University, 34000 Istanbul, Türkiye; kadriye.ozturk@bilecik.edu.tr; 2 Department of Statistics and Computer Sciences, Faculty of Science, Bilecik Seyh Edebali University, 11100 Bilecik, Türkiye; 3 Department of Statistics, Faculty of Arts and Sciences, Yildiz Technical University, 34220 Istanbul, Türkiye

**Keywords:** ESG forecasting, sustainable finance, information theory, probability calibration, forecast stability

## Abstract

Sustainable finance exhibits non-linear dynamics, regime shifts, and distributional drift that challenge conventional forecasting, particularly in volatile emerging markets. Conventional models, which often overlook this structural complexity, can struggle to produce stable or reliable probabilistic forecasts. To address this challenge, this study introduces a complexity-aware forecasting framework that operationalizes information-theoretic meta features, Shannon entropy (SE), permutation entropy (PE) and Kullback–Leibler (KL) divergence to make Environmental, Social, and Governance (ESG) index forecasting more stable, probabilistically accurate, and operationally reliable. Applied in an emerging-market setting using Türkiye’s ESG index as a natural stress test, the framework was benchmarked against a macro-technical baseline with a calibrated XGBoost classifier under a strictly chronological, leakage-controlled nested cross-validation protocol and evaluated on a strictly held-out test set. In development, the framework achieved statistically significant improvements in both stability and calibration, reducing fold-level dispersion (by 40.4–66.6%) across all metrics and enhancing probability-level alignment with Brier score reduced by 0.0140 and the ECE by 0.0287. Furthermore, a meta-analytic McNemar’s test confirmed a significant reduction in misclassifications across the development folds. On the strictly held-out test set, the framework’s superiority was confirmed by a statistically significant reduction in classification errors (exact McNemar *p* < 0.001), alongside strong gains in imbalance-robust metrics such as BAcc (0.618, +12.8%) and the MCC (0.288, +38.5%), achieving an F1-score of 0.719. Overall, the findings of the complexity-aware framework indicate that explicitly representing the market’s informational state and transitions yields more stable, well-calibrated, and operationally reliable forecasts in regime-shifting financial environments, supporting enhanced robustness and practical deployability.

## 1. Introduction

Financial markets are complex adaptive systems shaped by information flows and characterized by nonlinear dynamics such as abrupt regime shifts and volatility clustering [1,2,3,4]. The global ascendancy of sustainable finance, with sustainable investment assets under management reaching USD 30.3 trillion as of early 2022 [5], introduces an additional layer of complexity, with Environmental, Social, and Governance (ESG) criteria increasingly influencing capital allocation decisions [6]. Yet capital flows into ESG assets remain heterogeneous across regions and over time [7], a pattern that is especially pronounced in emerging markets where data limitations, policy uncertainty, and liquidity frictions coexist. Evidence from MSCI [8] suggests that firms managing social and governance risks more effectively tend to exhibit superior financial performance, reframing sustainable finance as a problem of uncertainty management under regime variability.

Against this backdrop, prior research has evolved along three broad lines: portfolio construction and optimization integrating ESG criteria [9,10], machine-learning applications for firm- and market-level prediction [11,12], and volatility/risk forecasting with advanced time-series models [13,14,15]. In practice, ESG index forecasting typically follows two established approaches [16]: fundamental analysis, which exploits macroeconomic and commodity-linked variables to capture structural drivers [17,18,19], and technical analysis, which leverages historical price patterns, momentum, and volatility as proxies for short-horizon dynamics [13,20]. Hybrid frameworks combine these inputs [15], yet most remain limited to feature aggregation and treat uncertainty and regime dependence implicitly, lacking mechanisms to represent distributional change or dynamical structure.

Information theory provides a principled framework for modeling financial time series as complex adaptive systems. It encompasses families of entropy, divergence, and dynamical complexity descriptors that quantify, respectively, the unpredictability of returns, shifts in the data-generating distribution, and the temporal organization of price dynamics. Implemented in a rolling, time-respecting manner, these descriptors explicitly capture regime dependence and distributional shifts in the return-generating process, complementing conventional fundamental and technical indicators [21,22].

This information-theoretic perspective is particularly well suited to the practical challenges of sustainable finance. Emerging ESG markets are prone to nonlinear dynamics triggered by policy uncertainty, regulatory interventions, and greenwashing controversies, all of which can induce abrupt regime shifts. Conventional models often struggle to identify such transitions. Information-theoretic measures such as Shannon Entropy (SE), Permutation Entropy (PE), and Kullback–Leibler (KL) divergence are explicitly designed to quantify distributional uncertainty, temporal complexity, and structural shifts. Collectively, they provide a principled quantitative framework for capturing informational instability and policy-induced transitions that characterize sustainability-linked financial systems.

Building on this rationale, the need for such explicit representations is heightened in the burgeoning field of sustainable finance: ESG indices exhibit methodology drift and high constituent turnover, while ESG capital flows are regime-sensitive and nonstationary. The challenge is particularly acute in emerging markets, where volatility couples with limited data depth and policy uncertainty. Although the literature on ESG index forecasting is limited, the explicit operationalization of information-theoretic descriptors within predictive pipelines remains scarce, underscoring a clear research gap in applying these tools to one of the most complex and increasingly important domains in modern finance.

This study introduces a complexity-aware forecasting framework by systematically augmenting a hybrid machine-learning pipeline with information-theoretic measures. The framework represents ESG index dynamics along two complementary dimensions: (i) distributional properties, where SE proxies for uncertainty and KL Divergence detects structural change; and (ii) dynamical structure, where PE captures the temporal and ordinal patterns in price dynamics. By integrating these signals alongside conventional macro-technical inputs, the design moves beyond simple feature aggregation to provide an explicit diagnostic of the market’s informational state and regime dependence. The central hypothesis is that such augmentation improves not only predictive accuracy but, more importantly, the stability and calibration of forecasts across heterogeneous regimes. Its practical utility and robustness were further demonstrated by a stress test in Türkiye’s highly volatile market, underscoring effectiveness in a challenging real-world setting and broader relevance to other emerging economies.

To rigorously evaluate the incremental value of information-theoretic features, this study implements a multi-phase, leakage-aware, and time-consistent framework. Phase 1 (Model and Data Preparation) defines the next-day direction target, constructs the competing baseline and entropy-augmented feature sets, and specifies a bespoke time-respecting calibrated XGBoost classifier. Phase 2 (Model Validation and Comparison) employs a forward-chaining nested cross-validation to jointly tune XGBoost hyperparameters and the probability-calibration component (Platt or isotonic);and to produce unbiased out-of-sample predictions for statistical testing at two granularities: at the fold level, robustness of performance differences (Wilcoxon signed-rank, Hodges–Lehmann) and stability (ΔCV% with BCa CIs and permutation tests); at the prediction level, probabilistic quality (Brier, ECE) and binary-decision agreement (McNemar’s test). Phase 3 (Final Hold-Out Evaluation) refits both specifications on the full development span under the selected configurations, fixes the decision threshold ex-ante from development data, and reports generalization on the strictly held-out test set using the pre-specified calibrator and threshold.

The remainder of this paper was organized as follows. Section 2 reviews the relevant literature to formally establish this research gap. Section 3 describes the data and the proposed methodological framework. Section 4 presents the empirical results, and Section 5 concludes the paper.

## 2. Literature

The ascendance of sustainable development as a global norm [23,24] has transformed financial thinking, prompting a re-evaluation of sustainability through its three core dimensions: economic efficiency, environmental protection, and social equity [23,25]. One of the key mechanisms for operationalizing this perspective is the ESG index, which serves as a benchmark for firm performance at the micro level and as a potential indicator of systemic resilience at the macro level, though this role remains debated [15]. Major providers such as S&P Global, Dow Jones, and MSCI have introduced widely used indices, including the Dow Jones Sustainability World Index and MSCI’s World and Emerging Markets ESG Focus series, which underpin investable products like ETFs from iShares, Vanguard, SPDR, and Fidelity [7,8,26]. Alongside these global benchmarks, national adaptations such as Türkiye’s BIST Sustainability Index, Brazil’s ISE B3, and South Africa’s JSE Responsible Investment Index reflect local macroeconomic priorities and regulatory contexts.

Against this backdrop, a vast and methodologically diverse academic literature has emerged to understand and model these complex assets. This section proceeds as follows: it first examines the multi-scale determinants that drive ESG performance, from macro-financial variables to firm-specific characteristics. It then critically evaluates the evolution of forecasting methodologies, from traditional econometrics to modern machine learning, highlighting their capabilities and limitations. Following this, it introduces information-theoretic concepts as a powerful alternative lens for capturing the complexity that other models overlook. Finally, it synthesizes these streams to identify a critical research gap and motivate the complexity-aware modeling framework proposed in this study.

### 2.1. Key Determinants of Financial Sustainability in ESG Markets: A Multi-Scale Perspective

Extensive research demonstrates that ESG performance is shaped by a multi-scale interplay of market dynamics, institutional structures, and firm-specific strategies. To provide a concise synthesis of this literature, Table 1 organizes the principal determinant categories into four analytical domains: (1) macro-financial and market, (2) institutional and structural, (3) corporate and firm-level, and (4) social–environmental and behavioral.

Within this framework, ESG outcomes emerge from complex and interdependent relationships across these domains rather than from isolated causal mechanisms. This documented complexity highlights the importance of developing forecasting methodologies capable of capturing non-linear, cross-level, and dynamic dependencies.

### 2.2. Traditional and Emerging Approaches to ESG/Sustainability Index Forecasting

The analytical challenges outlined above have driven the evolution of methodologies used to analyze and forecast ESG and sustainability indices. Over the past decade, approaches have gradually shifted from conventional econometric frameworks toward data-driven machine learning (ML) and deep learning (DL) paradigms. As researchers sought to capture the complex, non-linear, and interdependent dynamics of sustainability markets, this transition marked a methodological turning point from explanatory, assumption-driven models to flexible algorithms capable of uncovering hidden patterns and adaptive relationships within ESG and financial data.

Early studies predominantly relied on panel data analysis and regression-based methods, including GMM, quantile regression, and Structural Equation Modeling (SEM), among others [36,39,42,43,46,47,53]. While these approaches provided foundational empirical insights, their dependence on linearity and stationarity assumptions limited their ability to represent the non-linear complexity inherent in sustainability indices.

The most significant methodological advance has been the widespread adoption of ML and DL models, which shift from theory-driven to data-driven forecasting [56]. Early applications employed Decision Trees, Artificial Neural Networks, and Support Vector Machines [57,58], while ensemble methods such as Random Forest (RF) demonstrated superior predictive power in financial sustainability contexts [59]. More recent studies have combined ML with efficiency analysis frameworks, notably Data Envelopment Analysis integrated with RF and interpreted using SHAP analysis [60] and have developed specialized DL models for ESG-specific price and volatility prediction [9,20,61,62]. Among these, Long Short-Term Memory (LSTM), Convolutional Neural Networks (CNN), Gated Recurrent Units (GRU), and their hybrid variants are the most widely adopted architectures in financial forecasting [63]. Recent applications in ESG domains confirm their effectiveness, with studies demonstrating strong predictive performance for volatility and risk modeling [9,15,20,61,62].

To provide a synthetic overview of this rapidly expanding literature, Table 2 summarizes recent ESG forecasting frameworks, organizing them by methodological class, feature composition, and forecasting objective. The comparative synthesis reveals three key trends: a shift from single-model to hybrid architectures; an increasing integration of textual and unstructured information; and growing attention to interpretability and calibration through model-agnostic techniques such as SHAP or focal-loss optimization. Despite these notable advances in predictive accuracy and model flexibility, most existing studies still treat heterogeneous ESG features as static aggregates and lack a principled framework to quantify informational uncertainty, temporal complexity, and regime-dependent behavior. This methodological gap motivates the integration of entropy-based measures into forecasting frameworks.

### 2.3. Information-Theoretic and Entropy-Based Approaches in Financial Time Series Modeling

Conventional econometric and even many machine learning models often fall short when confronted with the strong non-linearities and regime-dependent patterns of financial markets. To address this limitation, information theory provides a principled lens, viewing markets as adaptive systems defined by uncertainty, complexity, and change [64].

Within this framework, SE remains a foundational measure of distributional uncertainty and informational diversity in financial markets [65,66], with recent applications to market efficiency and volatility analysis [67,68]. Moving beyond static uncertainty measures, PE captures the temporal organization of price dynamics through ordinal patterns, quantifying how the order of successive observations evolves over time [69,70,71]. This property enables PE to detect hidden regimes, local nonlinearities, shifts in market efficiency [72,73,74], and reveal instability through irreversibility analysis [75].

In contrast, KL divergence quantifies the distance between probability distributions, offering a complementary view of complexity by identifying structural breaks and regime transitions through changes in the overall shape of return distributions [76,77,78,79,80]. While PE is inherently sensitive to temporal order, KL divergence captures broader distributional reconfigurations, making both measures jointly powerful for modeling structural change and instability in financial systems.

Despite their proven value in detecting hidden complexity, distributional change, and systemic stress in traditional financial markets [77,81,82], these entropy-based tools remain underexplored within sustainability forecasting. This underutilization defines a crucial methodological gap that the present study seeks to address.

### 2.4. Synthesizing the Literature: The Case for a Complexity-Aware Forecasting Framework

The preceding literature review reveals a central paradox in sustainability finance. While the field has made important progress in identifying the multi-scale determinants of ESG performance and has increasingly adopted machine learning for forecasting, these advances have also exposed a deeper methodological gap: a disconnect between the complexity of ESG markets and the ability of existing models to capture their informational dynamics. Even sophisticated models tend to treat heterogeneous features as simple aggregates, without a principled way to quantify the emergent properties (uncertainty, complexity, and structural instability) that characterize sustainability-linked assets.

This study addresses this gap by proposing a complexity-aware forecasting framework. The core innovation lies in shifting from feature aggregation to the explicit modeling of market information. The framework systematically integrates a curated set of information-theoretic metrics: SE (distributional uncertainty), PE (sequential complexity), and KL divergence (structural shifts). Together, these measures allow the model to quantify both the state of the market and its transitions.

The central hypothesis is that this augmentation improves not only predictive accuracy but also, and more importantly, the stability and calibration of forecasts across heterogeneous regimes. Together, stability and calibration enhance a forecast’s practical value in volatile ESG markets: stability ensures consistent performance across different regimes, while calibration provides the quantifiable confidence necessary for granular risk management and reliable decision-making under uncertainty.

To demonstrate its external validity, the framework was applied to Türkiye’s BIST Sustainability Index, an emerging-market ESG index that serves as a natural stress test. The index operates in an emerging-market setting and is notably exposed to geopolitical shocks, abrupt policy changes, and volatility in cross-border capital flows. The framework’s ability to deliver robust gains in this stress-test setting highlights not only its practical relevance for emerging-market contexts but also its potential as a portable and generalizable methodology for embedding informational dynamics into predictive modeling across global sustainability finance.

## 3. Data and Methodology

This section presents an end-to-end, complexity-aware framework to forecast the next-day directional movement of the BIST ESG index. The study evaluated, under strict time-respecting and leakage-aware conditions, whether augmenting a baseline of macroeconomic and technical indicators with information-theoretic meta-features improved (i) predictive discrimination, (ii) probability calibration, and (iii) temporal stability of performance.

The workflow was organized as follows. Section 3.1 documents the dataset and the multi-stage feature-engineering protocol. Section 3.2 details the modeling pipeline, including the temporal data-partitioning scheme, the baseline versus information-theoretic augmented feature sets, and a calibrated classifier tailored to time-series classification. Section 3.3 presents the evaluation design, comprising nested cross-validation, a suite of statistical comparison procedures, and the final assessment on a strictly held-out test set.

### 3.1. Data

#### 3.1.1. ESG Index Data

The primary dataset is the BIST Sustainability Index (XUSRD), the first ESG benchmark launched by Borsa İstanbul on 4 November 2014 and widely recognized as Türkiye’s national ESG index. The index comprises firms listed on BIST that meet published ESG performance criteria, and its composition is reviewed on a regular schedule (typically annually), so the number of constituents varies over time.

For the empirical analysis, daily index levels were obtained from the Central Bank of the Republic of Turkey (CBRT) electronic data distribution system for the period 4 November 2014 to 5 November 2024. The dataset includes standard OHLC (Open, High, Low, Close) prices, recorded in TRY. These series formed the basis for all return calculations and the construction of the derived features used in the forecasting models.

#### 3.1.2. Complementary Market Variables

To capture the broader market environment in which the ESG index operates, a curated set of complementary variables was incorporated into the analysis. These macro-financial indicators were selected to represent both global market dynamics and local systematic risks. Specifically, three external variables were employed: the USD/TRY exchange rate, commonly regarded as an indicator of financial stability and exposure to external shocks in emerging markets [83]; the XAU/USD gold price, widely recognized as a proxy for global risk aversion and a safe-haven asset during episodes of financial stress [84]; and the Brent crude oil price, which reflects global economic activity and inflationary pressures [85]. Incorporating these variables enabled the framework to account for macroeconomic conditions in parallel with the endogenous dynamics of the ESG index.

#### 3.1.3. Technical Indicators

Technical indicators were incorporated into the feature set as quantitative proxies for market psychology and trading behavior. A substantial body of empirical research shows that such indicators, by capturing non-fundamental aspects of price formation, can enhance forecasting performance across both econometric and machine-learning approaches [86,87,88,89,90,91,92]. In this study, a curated set of indicators was employed and grouped into three functional categories: (i) trend-following measures (Exponential Moving Average (EMA) and Parabolic SAR-PSAR) to capture directional momentum; (ii) momentum oscillators (Relative Strength Index (RSI) and Williams %R) to assess the velocity of price movements and identify overbought/oversold conditions; and (iii) a volatility measure (Average True Range (ATR)) to quantify the scale of market fluctuations. The definitions, formulas, parameterizations, and expected roles of these indicators are summarized in Table 3.

### 3.2. Feature Engineering

Feature engineering in this study targets representations that complement the macroeconomic context by encoding endogenous ESG market dynamics. While macro variables summarize exogenous conditions, they are often insufficient to capture investor-driven regime shifts. Classical technical indicators (e.g., EMA, PSAR, RSI, Williams %R, and ATR) quantify trend, momentum, and volatility, but largely remain surface-level statistics of price paths. To address this representational gap, information-theoretic features (comprising market-state and market-transition indicators) were introduced as principled descriptors of systemic uncertainty, structural change, and sequential complexity in financial time series.

The construction of the final feature space followed a two-stage process. First, a leakage-aware time-series cross-validation procedure was used to optimize the lookback parameters of the classical technical indicators (Section 3.2.1). Second, the set of information-theoretic features was computed under the same time-respecting alignment (Section 3.2.2). Together with the block of raw macro-financial variables, these derived feature sets form the complete pool of predictors used in the modeling phase.

#### 3.2.1. Optimization of Technical Indicator Parameters

Rather than relying on default parameters, a data-driven procedure was applied to select lookback windows for indicators with tunable horizons. Grid searches were defined a priori for EMA∈ {10, 20, 30, 40, 50}), RSI ∈ {7,14,21}, ATR ∈ {10,14,20} and Williams %R ∈ {7,14,21}. PSAR was retained at conventional settings (acceleration factor starting at 0.02, incrementing by 0.02 on each new extreme, and capped at 0.20) to reflect standardized practice and avoid unnecessary overfitting.

The optimization protocol was designed to be model-agnostic and leakage-aware. For each candidate window, the univariate indicator series was evaluated against the binary next-day direction target using a 5-fold TimeSeriesSplit restricted to the training data. Discrimination was quantified by the Area Under the ROC Curve (AUC), which was directionally adjusted using the transformation max(AUC, 1—AUC). This ensures that the optimization captures the strongest predictive signal, regardless of whether the correlation is positive or negative. The window length yielding the highest mean validation AUC was selected as optimal for each indicator.

The resulting set of optimized horizons for EMA, RSI, ATR, and Williams %R with PSAR at conventional settings constituted the finalized technical-indicator configuration used downstream in the modeling pipeline, with all computations performed under time-respecting alignment (no look-ahead).

#### 3.2.2. Information-Theoretic Feature Extraction

To capture non-linear dynamics and informational properties that price-based technical indicators and macro-financial covariates may not fully encode, the feature set was augmented with information-theoretic meta-features. Following the information-theoretic tradition in econometrics and return predictability [64,76], these measures are not trading rules; rather, they serve as a diagnostic layer that quantifies market uncertainty (entropy), dynamical complexity, and susceptibility to structural change and regime shifts, thereby contextualizing both macroeconomic and technical signals [64,76,77].

Let $r_{t}=\left(P_{t}-P_{t-1}\right)/P_{t-1}$ denote the simple return of the ESG index closing price, where $P_{t}$ is the closing price at time t. Three information-theoretic features were extracted over a 14-day rolling window of $r_{t}$ to capture distinct aspects of the series’ informational properties. The selected features consist of market state indicators and a market transition indicator which are detailed below. For measures requiring discrete probability distributions, a discretization with K = 10 bins was employed; to ensure numerical stability, probabilities were offset by ε (=10^−12^) prior to logarithmic operations. Further operational details for SE/PE (state) and KL (transition) are provided in the subsections below.

*Market State Indicators: Entropy Measures*

The informational state of the ESG market was evaluated with two complementary entropy measures: SE, which captures distributional uncertainty, and PE, which reflects sequential (ordinal) complexity.

SE, a foundational metric introduced by Shannon (1948) [65], measures the average uncertainty of the return distribution within each rolling window. For a given window of simple returns, a discrete probability distribution ${\left\{p_{i}\right\}}_{i=1}^{K}$ was constructed from histogram-binned returns, where $\pi_{i}$ is the empirical probability of the $i^{th}$ bin. Conceptually, higher SE reflects a more dispersed and unpredictable market state, typically associated with elevated volatility and diverse investor expectations, whereas lower SE indicates concentrated and highly predictable returns with minimal informational diversity, conditions that often occur during periods of market consensus or one-sided trading phases. The mathematical formulation for SE was provided in Appendix A.

PE, a model-free measure introduced by Bandt and Pompe (2002) [69], quantifies the sequential (or ordinal) complexity encoded in the relative ordering of consecutive values rather than their magnitudes, making it robust to outliers [92] and suitable for noisy, non-stationary financial data (e.g., [81,82,103]). Conceptually, lower PE indicates a loss of informational diversity and an increase in synchronized trading behavior, conditions that are typically associated with herd dynamics and reduced market efficiency, whereas higher PE reflects more heterogeneous and adaptive market interactions.

To compute PE, the return series within each window, $\left\{r_{t}\right\}$, was mapped into overlapping vectors of length d = 3 (embedding dimension) with a time lag of τ = 1 [104]. Each vector was then assigned to one of the d! possible ordinal patterns (permutations). Let p($\pi_{j}$) denote the empirical frequency of a given permutation $\pi_{j}$. The normalized PE, which scales the entropy value to a range between 0 (perfectly ordered) and 1 (completely random), is formally defined in Appendix A.

*Market Transition Indicator: Divergence Measure*

While SE and PE were employed to quantify the within-window informational state, a between-window measure was required to detect structural changes in the return-generating process. For this purpose, the KL divergence was adopted as the primary market transition indicator. The choice of KL is motivated by several properties: its asymmetry is advantageous for modeling temporal processes; it is a standard metric in the econometrics of regime detection; and it remains computationally efficient in a rolling-window implementation [76,105,106].

For each rolling window, a discrete probability distribution ${P=\left\{p_{i}\right\}}_{i=1}^{K}$ was derived from the returns using the histogram method with K bins. The reference distribution ${Q=\left\{q_{i}\right\}}_{i=1}^{K}$ was constructed in the same manner from the immediately preceding window (shifted by one day). KL divergence measures the information change from the previous distribution Q to the current distribution P; higher values indicate potential regime shifts.

Economically, higher KL values indicate pronounced regime transitions or external shocks that disrupt the prevailing market structure and reflect a sharp increase in distributional instability and informational surprise between consecutive time windows. In contrast, lower KL values correspond to stable market conditions where the return-generating process evolves smoothly and remains consistent with recent history. The formal computation for KL divergence is provided in Appendix A.

The complete set of predictors used in the modeling phase is organized into three coherent categories. The macroeconomic variables were incorporated in raw level form without scaling or transformation. All technical indicators were computed directly from the OHLC price series, with PSAR retained at its standard parameterization and the lookback windows for EMA, RSI, ATR, and Williams %R selected through the leakage-free time-series cross-validation procedure. The information-theoretic features (SE, PE, and KL divergence) were derived from the daily simple-return series (pct change), and no normalization or scaling (e.g., z-score or min–max) was applied, in order to preserve the distributional characteristics essential for valid entropy- and divergence-based diagnostics. This finalized, category-structured feature set is summarized in Table 4, which provides a concise overview of all engineered predictors used in the subsequent modeling workflow.

### 3.3. Modeling Framework

The complexity-aware framework was designed for a fair, leakage-aware comparison between two specifications: a baseline model, comprising macro-financial variables and optimized technical indicators, and an information-theoretic-augmented model, which additionally incorporates SE, PE, and KL divergence features. These information-theoretic signals were treated as diagnostics of market state and transitions and were evaluated for their incremental contribution to probability calibration, fold-to-fold stability, and directional accuracy under a time-consistent design. The workflow proceeded in three main phases: (i) model and data preparation, (ii) validation and comparison strategy, and (iii) final model training and hold-out evaluation.

#### 3.3.1. Phase 1: Model and Data Preparation

**Figure 1:**
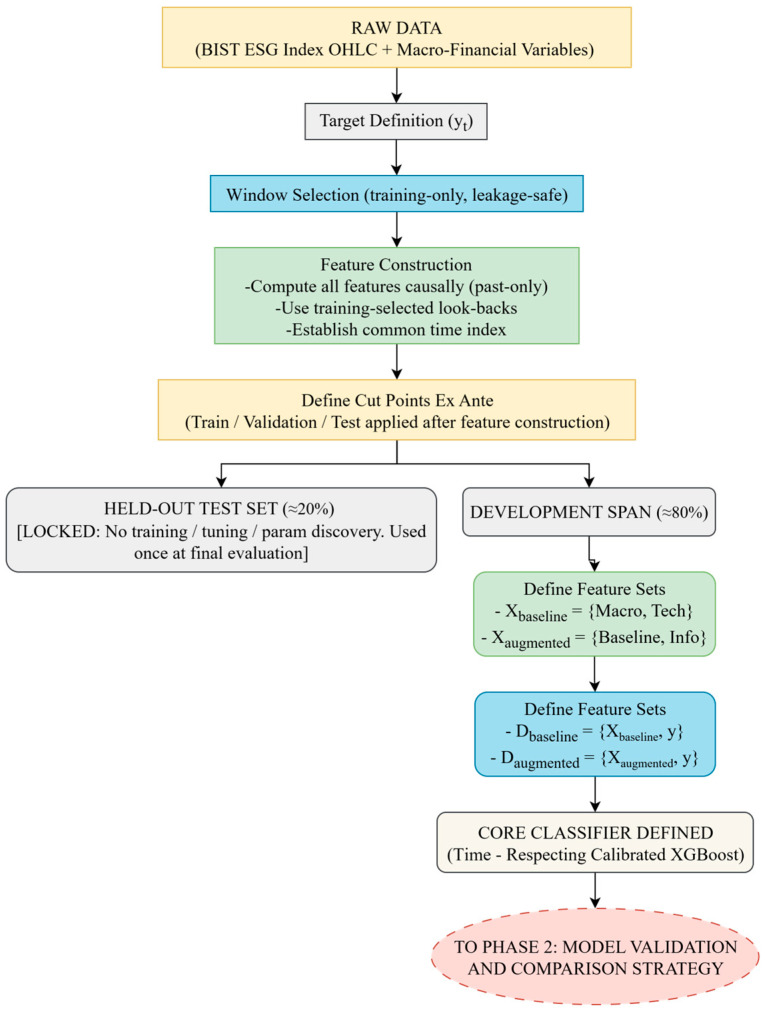
Schematic of the Phase 1 data preparation and partitioning workflow.

In this phase, the dataset was partitioned chronologically into training, validation, and a strictly held-out test block, and the prediction target was defined as the next-day direction. Two feature specifications were fixed: a baseline set and an information-theoretic-augmented extension adding entropy- and divergence-based measures. All features were computed causally, and the test block remained untouched. The same XGBoost classifier with a time-respecting probability calibration layer was specified for both feature sets. A schematic of this workflow is shown in Figure 1. The diagram illustrates the main data flow and feature construction steps leading from raw inputs to baseline and augmented model definitions under a time-respecting design.

*Temporal Data Partitioning*

To prevent look-ahead bias and to obtain an unbiased estimate of generalization performance, the dataset was partitioned chronologically into a development span (≈80%) and a strictly held-out test block (≈20%). Following common practice in financial time-series forecasting, the development span was further divided into a training block (≈60%) and a validation block (≈20%).

The roles of these subsets were strictly defined. The training set was used for initial model fitting and for leakage-aware optimization of technical indicator look-back windows, selected exclusively from training data. The validation set supported model selection and hyperparameter tuning within the development span.

To ensure temporal integrity and consistency, feature engineering was applied prior to the final partitioning. Features were computed causally (using only past information) to establish a common time index, which inherently handled the removal of warm-up periods. The definitive chronological split was then applied to this aligned, feature-rich dataset, ensuring that all subsets fully preserved the causal structure of the data. The test block remained strictly untouched throughout all development phases and was used only once to report the final out-of-sample performance.

*Target Construction*

The modeling task was formulated as a binary classification problem. The target variable $y_{t}$ was set to 1 if the next day’s closing price $P_{t+1}$ exceeded the current day’s closing price $P_{t}$, and 0 otherwise. This binary setup formalizes the task as predicting whether the next-day closing price will rise relative to the current day.

*Baseline and Augmented Feature Sets*

To isolate and evaluate the incremental contribution of the information-theoretic features across multiple dimensions of model performance, two distinct feature sets (design matrices) were constructed for a direct, like-for-like comparison. This evaluation was designed to assess the impact on not only directional accuracy but also on fold-to-fold stability and probability calibration.

$X_{\mathrm{baseline}}$: This specification includes only traditional predictors:

(i) The block of external macroeconomic variables;
(ii) The block of empirically optimized technical indicators and the fixed-parameter PSAR. No information-theoretic measures are included.

$X_{\mathrm{augmented}}$: This specification extends $X_{\mathrm{baseline}}$ with the full suite of information-theoretic measures, categorized as:

(i) The block of market state indicators (SE and PE);
(ii) The market transition measure (KL divergence).

Formally, let $X^{macro}$, $X^{tech}$, and $X^{info}$ denote the macroeconomic, technical, and information-theoretic blocks, respectively. The two design matrices are defined as:

$$X_{\mathrm{baseline}}=\left[X^{macro},X^{tech}\right],\;\;\;X_{\mathrm{augmented}}=\left[X^{macro},X^{tech},X^{info}\right].$$

Both specifications were evaluated under an identical protocol on the same temporal partition defined previously (≈60–20–20). Because the learner is tree-based and invariant to monotonic transformations, no feature scaling was applied; therefore, the shared predictors in $X_{\mathrm{baseline}}$ and $X_{\mathrm{augmented}}$ have identical values. Consequently, any observed performance difference is attributable solely to the inclusion of $X^{info}$. This experimental design yields two parallel datasets for the subsequent modeling phases:

$$D_{baseline}=\left\{X_{\mathrm{baseline}},y\;\right\},\;\;\;D_{augmented}=\left\{X_{\mathrm{augmented}},y\;\right\}$$

*Core Classifier: Time-Respecting Calibrated XGBoost*

The core predictive engine for both datasets defined previously, $D_{baseline}$ and $D_{augmented}$, was an XGBoost classifier, selected for its state-of-the-art performance on tabular data and built-in regularization against overfitting [107]. However, a known limitation of such classifiers is their tendency to produce miscalibrated probabilities [108]. As this study evaluated the models not only on classification accuracy but also on the quality of probabilistic forecasts using calibration-sensitive metrics (e.g., Brier score and Expected Calibration Error-ECE), addressing this issue was paramount.

To this end, a time-respecting calibration wrapper was implemented around the XGBoost classifier and applied identically to both D_baseline_ and D_augmented_. Calibration was performed after the initial model training, not before, to prevent any look-ahead bias, following standard practice in probability calibration (e.g., [108]), where calibration is applied post-training to adjust output probabilities without affecting the base learner. The process was nested within validation: in each outer fold of the nested CV, the calibration split followed the training split in time, and the mapping g was fit only on the scores obtained from this later, non-overlapping data. This protocol enforced causality by ensuring that the calibration step used past-only information relative to its application, thereby eliminating look-ahead and transforming raw scores into well-calibrated probabilities.

The calibration procedure followed a time-respecting, post-training design, ensuring that only past information was used at each step. Specifically, the development span was partitioned into a disjoint, past-only calibration holdout slice: the base learner (f) was trained on the earlier portion of the data, and the mapping function (g) was subsequently fitted on the scores obtained from this later, non-overlapping holdout. This approach enforced causality by calibrating model outputs strictly on future-unseen data within each fold. The formal algorithmic steps and mathematical definitions for this time-respecting calibration procedure are detailed in Appendix B (Time-Respecting Calibration Protocol).

Two well-established alternatives were considered for the mapping function g: Platt Scaling (logistic regression) [109], and isotonic regression (pool-adjacent-violators) [110]. To stabilize calibration, the fraction α was drawn from the range [0.15, 0.30), with a minimum of 100 observations enforced for the calibration split; potential overfitting of isotonic regression was mitigated by applying it only on temporally subsequent holdouts within inner validation. Class imbalance in each training split was handled by setting scale_pos_weight dynamically. A temporal gap equal to one feature-window length was enforced between train and validation segments in both inner and outer splits to avoid overlap-induced leakage. The choice of g and its parameters was treated as a tunable option within the development span and applied identically to both feature specifications $X_{\mathrm{baseline}}$ and $X_{\mathrm{augmented}}$, providing a consistent foundation for the validation and comparison procedures described in Phase 2.

#### 3.3.2. Phase 2: Model Validation and Comparison Strategy

**Figure 2:**
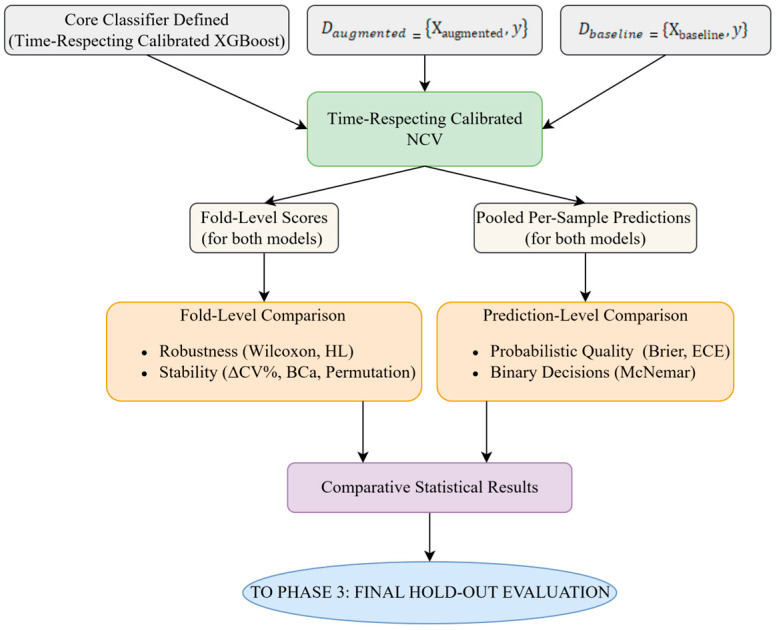
Schematic of the Phase 2 nested cross-validation and model-comparison workflow.

This phase details the protocol for validating and comparing the baseline and the information-theoretic augmented models on the development span (≈80%). The core is a time-respecting NCV in which the Time-Respecting Calibrated XGBoost classifier was tuned and evaluated. Applied identically to both datasets, the NCV used forward-chaining TimeSeriesSplit (3 inner folds, 5 outer folds) with strictly chronological splits (no shuffling, past-only information). The inner loop jointly tuned XGBoost hyperparameters and the probability-calibration component (Platt or isotonic), while the outer loop provided unbiased performance estimates. For each model, the procedure yielded (i) five outer-fold scores and (ii) a pooled set of out-of-fold, per-sample predictions from the calibrated classifier. These outputs underpinned two complementary analyses: fold-level comparisons (stability/robustness) and prediction-level comparisons (probabilistic quality and binary-decision agreement). The held-out test set was not used in this phase. A schematic of this workflow is shown in Figure 2, illustrating the NCV structure used for model tuning and comparison. The diagram highlights the generation of fold-level and prediction-level outputs under identical, time-respecting conditions.

*Nested Cross-Validation Protocol for Hyperparameter Tuning and Performance Estimation*

To identify the optimal configuration and obtain an unbiased estimate of generalization, a time-respecting NCV procedure was implemented on the development span (the first ≈80% of the sample), leaving the remaining ≈20% as a strictly held-out test block. The protocol was designed to preserve chronological order and prevent data leakage. Its main steps are summarized below.

1. Fold Structure
  A TimeSeriesSplit scheme was employed, which preserved chronological order (no shuffling) with 3 inner folds (for hyperparameter tuning via RandomizedSearchCV) and 5 outer folds (for performance estimation). The choice of a 3 × 5-fold structure aimed to balance the bias–variance trade-off, consistent with established recommendations for time-series tasks of this scale [111,112].
2. Inner Loop (Hyperparameter Tuning)
  Given the broad hyperparameter space of the XGBoost + calibration wrapper (see Supplementary Table S1), the inner loop used RandomizedSearchCV rather than an exhaustive grid due to the wide parameter ranges and the diminishing returns of exhaustive enumeration. Within this loop, the search treated the choice of calibration method (Platt vs. isotonic) as a tunable hyperparameter, which was optimized jointly with the standard XGBoost hyperparameters and a calibration-holdout fraction drawn from the range [0.15, 0.30), ensuring a minimum of 100 observations in the calibration slice. With a budget of n_iter = 200 per inner loop and a 3-fold TimeSeriesSplit, each outer fold evaluates approximately 600 candidate model fits; across 5 outer folds this totals approximately 3000 inner-loop fits per specification (200 × 3 × 5), plus 5 refits of the selected configurations.
3. Outer Loop (Performance Estimation)
  For each outer split, the model was trained on the outer-train slice, calibrated on its past-only calibration slice, and evaluated on the outer-validation slice. The resulting outer-fold scores were then aggregated to obtain an unbiased estimate of generalization performance.
4. Protocol Application and Bias Prevention

The same time-respecting NCV design (chronological splits, no shuffling) and search protocol were applied identically to both the baseline and the augmented specifications. By strictly insulating hyperparameter (and calibrator) selection from performance estimation, this nested design prevents optimistic bias and yields a robust, unbiased distribution of expected out-of-sample performance under identical experimental conditions.

*Performance Evaluation Metrics*

Let $y_{t}\;\in \left\{0,1\right\}$ denote the true label and ${\hat{p}}_{i}\;\in \left[0,1\right]$ the predicted probability for instance i. Confusion-matrix entries are denoted by TP (true positive), FP (false positive), TN (true negative), FN (false negative).

Directional accuracy metrics quantify thresholded classification performance and are robust to class imbalance. The suite includes: Balanced Accuracy (BAcc), which averages sensitivity (recall) and specificity; the F1-Score, the harmonic mean of precision and recall, which emphasizes positive-class detection; and the Matthews Correlation Coefficient (MCC), a robust correlation measure between predicted and observed classifications on a scale of −1 to +1. The formal definitions for directional acccuracy metrics (BAcc, Precision, Recall, F1, MCC) are provided in Appendix C.

Discrimination metric, the receiver operating characteristic area under the curve (ROC AUC) quantifies threshold-independent discrimination: it is the probability that a randomly chosen positive instance receives a higher score ${\hat{s}}_{i}$ (${\hat{p}}_{i}$) than a randomly chosen negative one (1.0 = perfect, 0.5 = random), i.e., the proportion of correctly ordered positive–negative pairs. Given the mild class imbalance, the F1-score was pre-specified as the primary evaluation metric, with BAcc, MCC, and ROC-AUC reported as complementary metrics for directional accuracy and discrimination. The formal definition for AUC is provided in Appendix C.

Probability calibration metrics quantify the alignment between a model’s probabilistic forecasts and the observed outcomes, independently of any decision threshold. The Brier score measures this via the mean squared difference between predicted probabilities and realized labels [113]. The Expected Calibration Error (ECE) summarizes the disparity between a model’s mean confidence and the empirical event rate within probability bins (here K = 10, equal width). Let $B_{k}$ denote bin k with size $n_{k}=\left|B_{k}\right|$ and $n=\sum_{k=1}^{K}n_{k}$ [114]:

(1) $$Brier=\frac{1}{n}\sum_{i=1}^{n}({\hat{p}}_{i}-y_{i}{)}^{2}$$

(2) $$ECE=\sum_{k=1}^{K}\frac{n_{k}}{n}\left|\frac{1}{n_{k}}\sum_{i\in B_{k}}y_{i}-\frac{1}{n_{k}}\sum_{i\in B_{k}}{\hat{p}}_{i}\right|$$

Model stability metrics quantify the consistency of performance across repeated estimates (e.g., outer folds) by measuring the dispersion of the performance scores, rather than their absolute magnitude. Let $m_{1},\ldots ,m_{k}$ denote the set of metric values over K repeats, with mean $\bar{m}$ and standard deviation s. Two summary statistics are defined to assess stability: the coefficient of variation (CV%) and a performance-to-stability ratio R; lower CV% (as it reflects lower dispersion relative to the performance level) and higher R (as it reflects a stronger signal relative to its noise) indicate greater stability.

(3) $$CV\%=100\times \frac{s}{\left|\bar{m}\right|}\;,\;\;\;\;\;\;\;\;R=\frac{\bar{m}}{s}$$

*Statistical Comparison of Model Performance*

This section details the statistical procedures used to test whether the augmented specification provided a significant improvement over the baseline and whether such gains were robust and stable. All comparisons used outer-fold predictions from the time-respecting NCV.

Fold-Level Comparison (Robustness and Stability)

Fold-level comparisons were conducted on the five outer-fold, out-of-sample scores from the calibrated XGBoost to assess both the robustness of performance differences and comparative stability.

First, robustness of performance differences was assessed at the fold level for the probability-calibration metrics (Brier score and ECE). The question was whether one specification exhibited a consistent advantage across the five calibrated NCV outer folds. Let $M_{f}^{\left(Aug\right)}$ and $M_{f}^{\left(Base\right)}$ be the augmented and baseline scores on outer fold f (=1,…,5), respectively, and define the per-fold difference as: ${∆}_{f}=M_{f}^{\left(Aug\right)}-M_{f}^{\left(Base\right)}$.

Statistical significance of the paired differences ${\left\{{∆}_{f}\right\}}_{f=1}^{5}$ was assessed using the Wilcoxon signed-rank (Pratt) test, complemented by the Hodges–Lehmann (HL) median effect [115] with a 90% bias-corrected and accelerated (BCa) bootstrap confidence interval [116], based on B = 20,000 bootstrap resamples and a paired sign-flip permutation test obtained by randomly swapping the augmented and baseline labels within folds.

Second, models’ stability was assessed to quantify and test differences in performance consistency between the baseline and augmented specifications, using directional-accuracy and discrimination metrics (BAcc, F1, MCC, ROC AUC). The question was whether one specification exhibited a statistically significantly lower variability (i.e., higher stability) across the five calibrated NCV outer folds. For each metric, stability was summarized as CV% computed over the five-fold-level scores for each model, and the stability contrast was defined as: $∆CV\%={CV\%}^{\left(Aug\right)}-{CV\%}^{\left(base\right)}$.

Additionally, to synthesize performance level and stability into a single, intuitive signal-to-dispersion ratio (where higher is better), the R metric was also computed and the reliability contrast between models was then defined as the difference in their respective R values: $∆R=R^{\left(Aug\right)}-R^{\left(base\right)}$.

Uncertainty for both ∆CV% and ∆R was quantified with a BCa confidence intervals based on B = 20,000 bootstrap resamples.

Prediction-Level Comparison (Paired, Sample-wise)

To complement the fold-level analyses, prediction-level comparisons were conducted on the pooled out-of-sample predictions from the five outer folds of the time-respecting NCV. For each observation, calibrated probabilities and the induced 0/1 decisions from the augmented and baseline specifications were paired, permitting per-sample evaluation of calibration quality and binary decision outcomes.

Out-of-sample predictions from all five outer folds were pooled, and calibrated probabilities from the two specifications were compared on a per-observation basis. For the Brier score, per-sample paired differences were formed as $d_{i}={\left({\hat{p}}_{i}^{\left(Aug\right)}-y_{i}\right)}^{2}-{\left({\hat{p}}_{i}^{\left(Base\right)}-y_{i}\right)}^{2}$, where negative values favor the augmented specification. The vector $\left\{d_{i}\right\}$ was evaluated using the Wilcoxon signed-rank test (Pratt variant); the HL median effect was reported. Uncertainty was summarized with a BCa bootstrap confidence interval (B = 20,000, index-bootstrap resampling), and a paired sign-flip permutation *p*-value was also computed for the mean difference.

For calibration error, ECE with K = 10 equal-width bins was computed on the same pooled predictions for each model, and the scalar contrast $∆ECE={ECE}_{K=10}^{\left(Aug\right)}-{ECE}_{K=10}^{\left(Base\right)}$ was assessed via BCa confidence intervals (B = 20,000, index-bootstrap); statistical significance was concluded when the interval excluded zero.

Finally, to compare paired 0/1 predictions, a fold-stratified McNemar’s test was employed. This approach was chosen over a standard pooled test to explicitly assess whether one model exhibited a consistently superior error profile across the different time periods represented by the five NCV outer folds. For fold f with index set $D_{f}$, let ${{\hat{y}}_{i}}^{\left(Aug\right)},{{\hat{y}}_{i}}^{\left(base\right)}\in \left\{0,1\right\}$. Define discordant counts as:

(4) $$n_{01}^{\left(f\right)}=\left|\left\{i\in D_{f}:{{\hat{y}}_{i}}^{\left(Aug\right)}=1\wedge \;{{\hat{y}}_{i}}^{\left(base\right)}=0\right\}\right|\;n_{10}^{\left(f\right)}=\left|\left\{i\in D_{f}:{{\hat{y}}_{i}}^{\left(Aug\right)}=0\wedge \;{{\hat{y}}_{i}}^{\left(base\right)}=1\right\}\right|$$

Exact two-sided McNemar tests were computed per fold to test ${H_{0}:n}_{01}^{\left(f\right)}=n_{10}^{\left(f\right)}$ (no asymmetry in error rates). Fold-specific *p*-values were then combined using Stouffer’s Z (equal weights) to obtain a single meta-analytic *p*-value, thereby preserving the NCV block structure and avoiding pooling [117,118].

#### 3.3.3. Phase 3: Final Model Training and Hold-Out Evaluation

This phase evaluated the baseline and the information-theoretic augmented models on the strictly untouched hold-out test block (final ≈ 20%). All design and tuning decisions were determined exclusively on the development span (first ≈ 80%) using time-respecting procedures.

**Figure 3:**
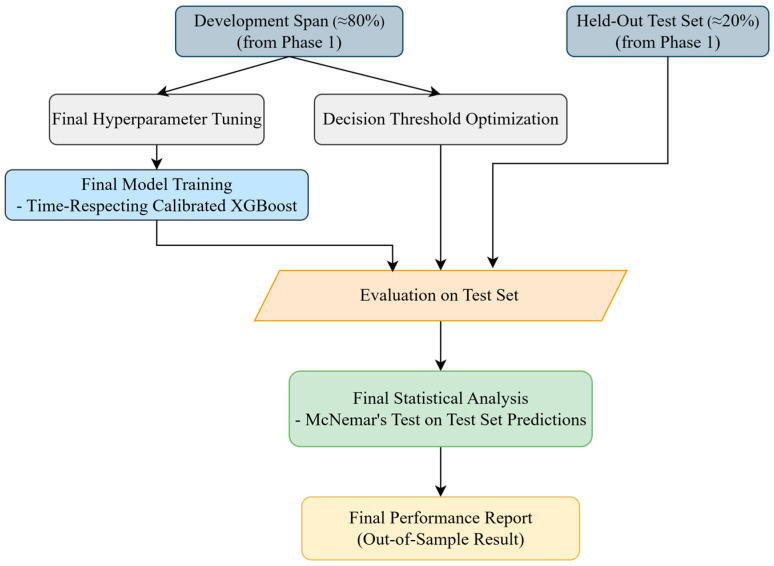
Schematic of the Phase 3 final training and hold-out evaluation workflow.

A schematic of this workflow is shown in Figure 3, which outlines the final training and evaluation stage on the strictly held-out test set, including the calibrated model refit, decision-threshold optimization, and McNemar’s statistical comparison of out-of-sample predictions.

For each specification, a time-respecting, calibrated XGBoost classifier was re-tuned on the development span with a forward-chaining TimeSeriesSplit (3 inner folds) and RandomizedSearchCV. The hyperparameter search space, CV scheme, scoring, and calibration protocol (isotonic or Platt) were identical to Phase 2.

Using the selected configuration, each model was refit on the full development span with a time-respecting calibration scheme: the base learner was trained on an earlier slice, and a held-out past-only slice within the development span was used to fit the chosen calibrator. The hold-out test block remained strictly untouched. Decision thresholds were fixed a priori on a per-model basis from out-of-fold calibrated probabilities obtained via forward-chaining CV on the development span, using a dense grid (0.05–0.95; step 0.005) to maximize F1. With calibrated probabilities and the frozen threshold, performance was then computed on the untouched test block.

Paired comparison on the hold-out test set was employed McNemar’s exact test (two-sided, exact binomial). Discordant counts (n_01_ and n_10_) and the resulting *p*-value were reported. Thresholds and the calibrator were pre-specified from development data; no test-set tuning was performed.

## 4. Results

### 4.1. Experimental Setup and Data Overview

Daily ESG data from 4 November 2014 to 5 November 2024 were analyzed. The raw price series contained no internal missing values and was transformed into daily simple returns, which served as the basis for all entropy and divergence computations. As the return series reflects the natural variation and occasional volatility characteristic of financial markets, no outlier filtering or winsorizing was applied. The only rows removed were the warm-up observations generated by rolling-window operations for entropy and technical indicators. Following these preprocessing steps, the aligned panel comprised 2642 trading days. For out-of-sample assessment, the chronologically last 529 days (~20%) were retained as a strictly held-out test block, while the preceding 2113 days (~80%) constituted the development span. All analyses respected the time order to avoid look-ahead bias (i.e., to ensure a leakage-free evaluation).

The daily simple-return series was examined for stationarity, trend, and serial dependence to characterize the underlying data-generating process. As shown in Table 5, an Augmented Dickey–Fuller test strongly rejected the unit-root hypothesis (*p* < 0.001), indicating stationarity. A Kendall–Tau test detected a mild but statistically significant positive monotonic trend. Ljung–Box statistics showed no joint autocorrelation at short horizons (lag 10), but confirmed significant serial dependence at longer lags (lag 20 and lag 50). The next-day direction target exhibited mild class imbalance (54% up vs. 46% down). Together, these observed behaviors of the ESG return series, namely weak-form stationarity together with a significant monotonic trend and long-horizon dependence, motivate the adoption of a predictive modeling framework.

The baseline feature set consisted of 3 macro-financial indicators (USD/TRY, XAU/USD, Brent) and 5 technical indicators (EMA, RSI, ATR, Williams %R, PSAR). The augmented model additionally included 3 information-theoretic features (PE, SE and KL divergence), yielding a total dimensionality of *p* = 11. Leakage-aware, time-respecting optimization selected EMA = 20, RSI = 21, ATR = 14, Williams %R = 21, while PSAR is non-windowed.

**Figure 4:**
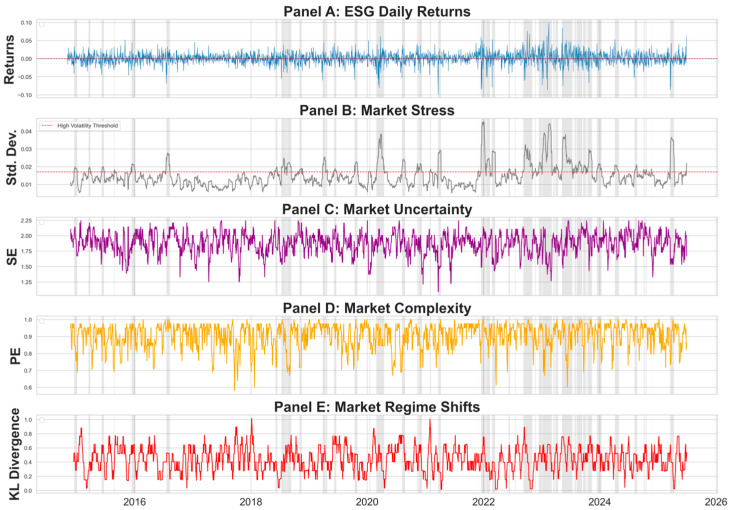
Diagnostic overview of ESG daily returns and information-theoretic signals.

Figure 4 provides a diagnostic overview of ESG daily returns and associated information-theoretic signals, each computed on 14-day rolling windows.

- Panel A shows a weakly stationary yet volatility-clustered return process, consistent with the ADF and Ljung–Box test results discussed in Section 4.1.
- Panel B presents the volatility, where shaded regions denote persistent high-volatility regimes, most prominently during the 2020 COVID-19 shock and the 2022–2023 turbulence period. The dashed line marks the high-volatility threshold, defined as the 75th percentile of the rolling volatility distribution.

The subsequent panels demonstrate how these information-theoretic signals act as empirical proxies for these market regimes, validating the motivation for their inclusion:

- Panel C reveals that SE tends to decline during and immediately after sharp market drawdowns (e.g., 2020), suggesting a temporary compression of informational diversity and a transition toward more consensus-driven, one-sided trading.
- Panel D shows that PE tends to decline in parallel with SE during high-stress episodes, illustrating its sensitivity to synchronized trading activity. As market stress (Panel B) intensifies, price dynamics appear to simplify and lose ordinal complexity, indicating the emergence of coordinated market movements and herd-driven behavior, conditions that are typically associated with diminished informational diversity and reduced market efficiency.
- Panel E shows that KL values often rise sharply during and immediately after major shocks (e.g., 2020, 2022), indicating its usefulness as a sensitive indicator of market regime transitions. The measure appears to capture both the intensity of structural breaks and the lingering distributional instability that can remain once the market’s underlying return-generating structure has been affected by external forces

Taken together, these diagnostics suggest that SE, PE, and KL function not merely as abstract statistical constructs but as empirically grounded indicators of distinct market conditions. SE reflects periods of market consensus or panic, PE captures patterns of herd behavior and reduced efficiency, and KL signals episodes of structural adjustment or external shocks. This interpretation provides a coherent and empirically supported rationale for including these measures in the augmented predictive framework.

### 4.2. Comparative Performance in Nested Cross-Validation

#### 4.2.1. Overall Performance Summary

The primary results for the baseline and information-theoretic augmented models, obtained under 5-fold time-respecting nested cross-validation with calibrated probabilities, are summarized in Table 6 (mean ± SD across outer folds). The augmented model attains higher mean BAcc (+0.0175; +2.8% relative), MCC (+0.0196; +7.1% relative), and ROC AUC (+0.0186; +2.7% relative), while the mean F1-score is comparable (0.6648 vs. 0.6646). Importantly, dispersion across folds is lower for the augmented model on every metric, with standard-deviation reductions of approximately 66.6% (F1), 50.1% (BAcc), 57.7% (MCC), and 40.4% (ROC AUC), indicating a more stable out-of-sample profile.

**Figure 5:**
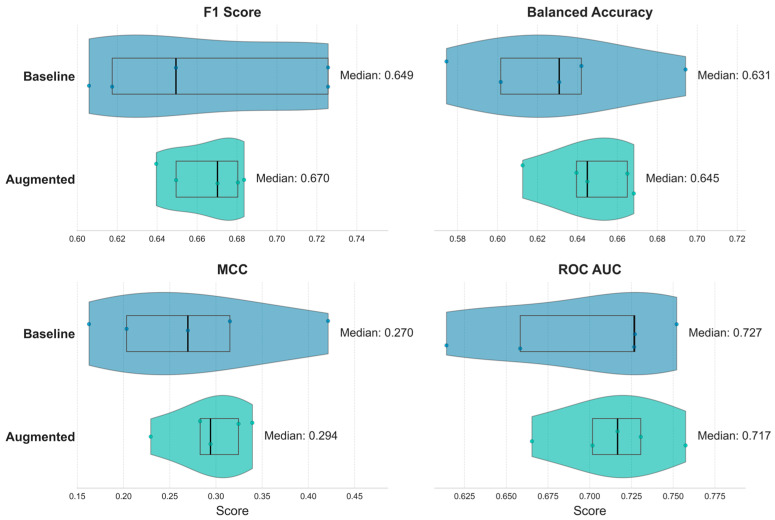
Fold-level raincloud plots of NCV performance metrics.

Fold-level distributions (Figure 5) corroborate the tabulated patterns, primarily highlighting the superior stability of the augmented model. Across metrics, the augmented specification yields tighter violins and narrower interquartile ranges, with fewer extreme values. For F1-score, although the means are similar in Table 6 (0.6648 vs. 0.6646), the median is higher for the augmented model (0.6702 vs. 0.6494), and its fold-to-fold spread is markedly smaller. For BAcc and MCC, both the center (e.g., medians around 0.645 vs. 0.631 for BAcc; 0.294 vs. 0.270 for MCC) and the dispersion favor the augmented model. For ROC AUC, the baseline median is slightly higher (0.7266 vs. 0.7167), yet the augmented model achieves a higher mean in Table 6 (0.7143 vs. 0.6957) and substantially lower variability, indicating a more reliable out-of-sample profile. Taken together, Figure 5 and Table 6 indicate that information-theoretic augmentation improves central performance on average and, more importantly, delivers consistently tighter fold-level distributions under identical NCV conditions.

#### 4.2.2. Statistical Significance of NCV Results

To assess robustness, fold-level differences in calibration quality were examined for Brier score and ECE across the five outer folds (Table 7). For both metrics, the HL estimator of the fold-level difference Δ = Aug − Base is negative, indicating lower error under the augmented specification. The BCa bootstrap confidence intervals for HL do not include zero (Brier: [−0.02784, −0.00610]; ECE: [−0.06678, −0.01868]), providing evidence consistent with improved calibration. Exact Wilcoxon signed-rank tests (two-sided) yield *p* = 0.0625 for both metrics, offering suggestive evidence at the 10% significance level, which is expected given the small sample (five folds) and the discrete nature of the test statistic. Taken together, these results indicate that, under calibrated predictions within the NCV protocol, the augmented model delivers more reliable probability forecasts across folds, with lower Brier and ECE on average.

To assess stability, variability across outer folds was quantified by CV% for each performance metric, and fold-level contrasts (ΔCV%) were formed between the augmented and baseline specifications (Table 8). Across all four metrics, ΔCV% was negative with BCa confidence intervals excluding zero, indicating that the augmented model exhibited consistently higher stability (lower fold-to-fold variability).

To complement this variability-focused measure, a performance-to-stability ratio R was also computed (Table 9), capturing performance per unit dispersion. Across all metrics, the augmented specification achieved substantially higher R values, more than doubling relative to the baseline for F1, BAcc, and MCC, confirming that improvements arise not only in average level but also in the reliability of performance across folds.

*Prediction-Level Comparison*

To assess calibration at the prediction level, pooled out-of-sample predictions from the five outer folds were analyzed. Using calibrated probabilities paired per observation (within fold), effects were evaluated for both probabilistic accuracies. As shown in Table 10, the augmented model achieved a lower mean Brier loss (Δ = −0.0140), with the BCa CI [−0.0199, −0.0084] excluding zero; Wilcoxon signed-rank testing (*p* = 0.0037) and a paired sign-flip permutation test (*p* < 0.001) corroborated this improvement. With 10 equal-width bins, ΔECE was −0.0287 and the BCa CI [−0.0440, −0.0117] excluded zero, evidencing a robust reduction in calibration error.

Finally, to compare the final 0/1 predictions, a fold-stratified exact McNemar’s test was conducted. Exact *p*-values from the five NCV outer folds were combined via Stouffer’s method, yielding a single meta-analytic result *p* = 0.0402. This indicates that the augmented specification achieved a statistically significant and consistent reduction in misclassifications relative to the baseline.

### 4.3. Definitive Performance on the Held-Out Test Set

This section reports definitive out-of-sample performance on the strictly held-out test set. Both specifications were trained on the full development span with their optimal hyperparameters. Decision thresholds were selected on out-of-fold calibrated probabilities from the development span to maximize F1 and then fixed for the test evaluation (Baseline: 0.070; Augmented: 0.415). The augmented threshold lies closer to a neutral 0.5 cut, consistent with a more balanced operating point under calibrated scores, whereas the baseline required a notably low cut-off to attain its best F1. The final hyperparameters used in the held-out evaluation are reported in Supplementary Table S2.

As shown in Table 11, at these fixed operating points the augmented specification outperformed the baseline on the threshold-dependent classification metrics. The largest gains appeared in imbalance-robust criteria: BAcc improved from 0.5480 to 0.6180 (Δ = +0.0700, +12.8%), and MCC from 0.2080 to 0.2880 (Δ = +0.0800, +38.5%). F1 increased from 0.7060 to 0.7190 (Δ = +0.0130, +1.8%), reflecting a better precision–recall balance at the chosen operating point. ROC AUC, which is threshold-independent, remained essentially unchanged (0.7210 vs. 0.7230; Δ = +0.0020, +0.3%). Calibration measures were broadly comparable across models (Brier ≈ 0.21; ECE ≈ 0.07).

The performance on the held-out test set confirms the patterns observed during cross-validation, supporting the overall robustness of the findings. While calibration quality is broadly comparable across models, the augmented specification demonstrates a clear advantage in discrimination, particularly in imbalance-robust metrics. This performance gain is statistically significant and practically meaningful, as shown by a paired McNemar’s exact test, which considers only discordant days in the out-of-sample period, that is, cases where the two models disagree, and evaluates whether one is systematically more accurate on the final predictions. The entropy-augmented model was correct on substantially more of these discordant observations (n01 = 58, n10 = 25; *p* < 0.001), indicating a higher conditional probability of correctness. Because the test window spans both tranquil and volatile ESG conditions, this asymmetric improvement suggests stronger generalization across changing market regimes rather than gains confined to stable periods.

### 4.4. Model Interpretability (SHAP Analysis)

**Figure 6:**
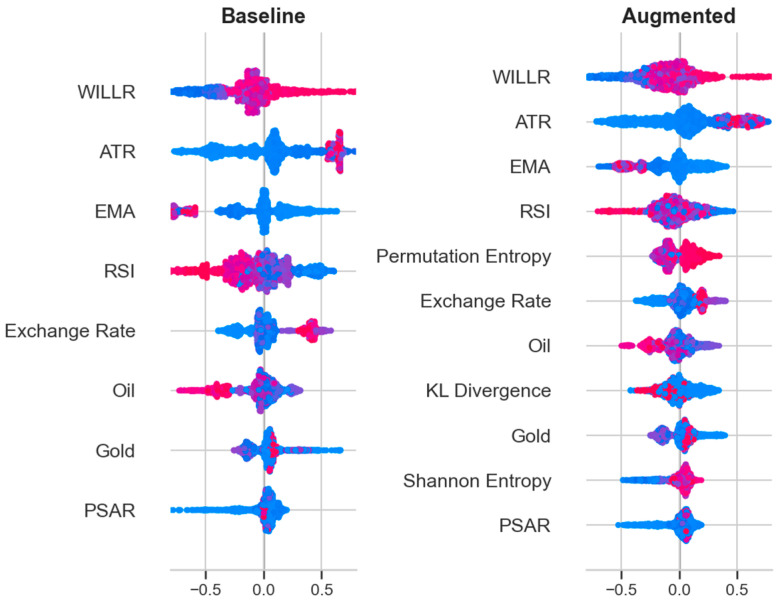
SHAP summary plots for the baseline and augmented models.

To enhance interpretability and assess the directional influence of each predictor on the next-day ESG index movement, a formal interpretability analysis was conducted using SHAP (SHapley Additive exPlanations). This method quantifies the magnitude and direction of each feature’s contribution to the final, calibrated model output. Figure 6 presents comparative SHAP summary plots for both the baseline and augmented models, computed on the full development span.

The SHAP-based interpretability analysis confirms Williams %R as the most influential predictor, with other technical indicators like ATR, EMA, and RSI also ranking as top contributors. These oscillators jointly capture momentum dynamics and exhibit a clear mean-reversion pattern: high values (overbought) are associated with negative SHAP contributions (predicting down), while low values (oversold) drive positive contributions (predicting up). Volatility measures (ATR) show symmetric, dispersed SHAP distributions, consistent with their role as risk amplifiers rather than direct directional signals. Macroeconomic variables (exchange rate, oil, gold) exhibit moderate, plausible effects; notably, exchange rate depreciation corresponds to downward ESG pressure, aligning with risk-off dynamics in the Turkish market.

Information-theoretic predictors show modest and theoretically consistent SHAP effects. Higher entropy values, indicating greater uncertainty, reduce the model’s confidence in an up forecast, while larger KL divergences capture structural regime shifts. Despite their lower marginal contributions, these information-theoretic variables enhance calibration and temporal stability, aligning with their theoretical role as information-state descriptors rather than direct return predictors.

### 4.5. Model Sensitivity (Entropy Window Parameter)

To test the framework’s temporal sensitivity, an entropy-window experiment was conducted under identical nested cross-validation and calibration protocols, varying the window length to capture different information regimes. The 7-day and 21-day configurations represent lower-window sensitivity and upper-window stability regimes, respectively. The former captures short-term, high-frequency dynamics with greater noise exposure, whereas the latter reflects more smoothed and stable structural patterns.

The NCV results for both entropy-window configurations (Supplementary Table S3) show that the entropy-augmented models consistently outperformed or matched their baseline counterparts under calibrated conditions, generally exhibiting reduced fold-to-fold variance and confirming the temporal robustness of the proposed framework.

Complementary robustness diagnostics (Tables S4 and S5) summarize fold-level calibration contrasts (Brier and ECE), stability measures (ΔCV %), and prediction-level calibration results. Across both alternative configurations (T = 7 and T = 21), the entropy-augmented models tended to achieve lower calibration errors and reduced fold-to-fold variability, particularly pronounced for the 21-day window, suggesting that the observed gains are systematic rather than incidental to the chosen window length.

The held-out test results further confirmed this pattern: the augmented model achieved higher BAcc under both settings (0.564 vs. 0.546 for T = 7 and 0.606 vs. 0.538 for T = 21), and McNemar’s exact tests (*p* = 0.0225 and *p* = 0.0032, respectively) indicated significant differences in directional classification favoring the augmented specification.

## 5. Discussion

Navigating the non-linear and regime-dependent dynamics of ESG markets poses a persistent challenge, particularly in emerging economies where volatility, liquidity constraints, and abrupt policy shifts are commonplace. This study introduces a complexity-aware forecasting framework that integrates information-theoretic diagnostics into a hybrid machine-learning design, computed in a strictly time-respecting (leakage-aware) manner. Applied to Türkiye’s BIST Sustainability Index as a natural stress test, the framework demonstrates that explicitly modeling the market’s informational state and transitions leads to more stable, better-calibrated, and more reliable forecasts under shifting market regimes. These results highlight the practical importance of capturing informational structure and regime dynamics, elements that are often overlooked by conventional macro-technical predictors, and they provide a solid foundation for the interpretive, theoretical, and applied insights presented in the following sections, as well as for the study’s concluding discussion of limitations and future research.

### 5.1. Interpretation of Findings

The augmented model’s advantage stems from its ability to represent the ESG market’s informational state and its transitions, aspects that conventional macro-technical predictors do not explicitly encode. Entropy- and divergence-based descriptors (SE, PE, and KL) condense key regime characteristics: uncertainty compression, ordinal simplification, and distributional shift. In practical terms, this means that the model perceives not only recent price movement but also how information itself is organized and transmitted through the market. By receiving signals aligned with regime change rather than raw volatility, the learner stabilizes its internal decision scale across heterogeneous conditions, which naturally enhances calibration and generalization.

From an economic perspective, this mechanism indicates that markets characterized by higher informational diversity (i.e., higher entropy) tend to exhibit more adaptive and resilient dynamics, as diverse information sources and heterogeneous expectations foster stability through decentralized adjustment. Conversely, periods of reduced or compressed entropy reflect phases in which market participants act upon similar information, leading to diminished informational heterogeneity and potential inefficiency. In this context, incorporating information-theoretic diagnostics into the modeling framework enables the learner to capture transitions from information-rich and stable regimes to stressed and synchronized ones, where predictive uncertainty becomes nonlinear. This ability allows the model to adapt to changing market conditions more realistically, improving both interpretability and calibration.

This interpretation is empirically supported by both the NCV and the held-out test results. Across validation folds, the augmented model achieved not only higher predictive accuracy but also markedly greater stability; its performance-to-stability ratio (R) more than doubled for key imbalance-robust criteria, indicating reliability gains far beyond simple level effects. Furthermore, the framework achieved statistically significant gains in probabilistic quality, producing better-calibrated forecasts at both the fold and prediction levels. This superiority was decisively confirmed on the strictly held-out test set, where the informational diagnostics led to a statistically significant reduction in misclassifications and concentrated its largest gains in the same imbalance-robust criteria (BAcc ≈ +12.8%, MCC ≈ +38.5%). Taken together, the evidence substantiates the central claim that incorporating information-theoretic diagnostics enhances not only point discrimination but also the stability, calibration, and interpretability of ESG index forecasts under heterogeneous and regime-dependent conditions.

### 5.2. Theoretical Implications

Theoretically, this study contributes to bridging the conceptual gap between information theory and sustainable finance by showing that entropy- and divergence-based measures represent a distinct and meaningful source of predictive information in ESG markets. Traditional macro-financial and technical indicators primarily reflect observable price dynamics but provide limited insight into the informational mechanisms that generate those dynamics. By conceptualizing ESG indices as complex adaptive systems in which agents interact under evolving beliefs, policy shocks, and heterogeneous information flows, the framework offers a reinterpretation of market predictability. It advances the perspective that predictability arises not solely from historical price patterns but from the market’s underlying informational diversity and structural adaptability.

Within this theoretical framework, the non-linear dynamics of ESG markets are not statistical noise to be filtered out but quantifiable reflections of the system’s adaptive state, challenging the assumptions of perfect informational efficiency. These information-theoretic diagnostics transform the abstract notion of market adaptability into measurable constructs that capture how information is organized, transmitted, and restructured over time. SE serves as a direct proxy for the market’s informational diversity and resilience: a market with high SE reflecting a broad distribution of beliefs, strategies, and expectations is typically more adaptive and resistant to shocks. Conversely, periods of declining SE and PE reveal a contraction in informational variety, often associated with consensus-driven or herd-like behavior. Such states of compressed entropy and high synchronization are informationally fragile and prone to the structural realignments that KL divergence is designed to detect. Collectively, SE, PE, and KL provide a coherent theoretical bridge linking market complexity, behavioral coordination, and structural change within sustainable finance systems.

Extending beyond the empirical context, entropy as a measure of informational richness represents a universal principle of complex adaptive systems rather than a market-specific artefact. Its theoretical foundation in information theory underpins its applicability across diverse financial environments. Nonetheless, the expression of these informational dynamics is shaped by contextual factors such as volatility, liquidity, and institutional structure that influence how information is assimilated and reorganized. In this sense, the emerging market context represented by the Turkish ESG index serves as a natural stress test. Its regime-shifting, high-volatility environment amplifies the informational signals that entropy metrics are designed to detect. The augmented model’s superior performance in this setting provides strong empirical evidence that these signals become most critical precisely when market complexity is high and conventional models falter. This is demonstrated not only by its asymmetric advantage in correctly classifying discordant predictions but also by its superior stability and calibration. This robustness under stress supports their generalizability and underscores their relevance for understanding market complexity in both emerging and developed economies.

### 5.3. Practical Implications

From a practical perspective, the proposed framework provides an implementable methodology for investors and policymakers, particularly in emerging ESG markets where volatility, policy shifts, and informational asymmetries are pervasive. Its practical utility lies in delivering forecasts that are not only demonstrably more stable across regimes but also better calibrated. This emphasis on calibration is particularly crucial; the quality of probabilistic predictions is a fundamental determinant of their reliability [119], and achieving accurate calibration remains a known challenge for modern, high-capacity classifiers like the one used in this study [120]. Beyond these forecasting improvements, the underlying information-theoretic diagnostics also serve as a complementary analytical layer. These measures help to illuminate the market’s underlying informational dynamics (e.g., shifts in consensus, herd-like coordination, or structural breaks), thereby offering a perspective that conventional indicators alone do not fully capture.

For investors and risk managers, the practical utility of this framework is empirically supported by its superior performance across both the NCV and the strictly held-out test set. The observed improvement, reflected in greater stability, better calibration, and fewer misclassifications on imbalance-robust metrics, results from the model’s ability to interpret underlying informational signals, providing a deeper contextual understanding than price-based indicators alone. Accordingly, investors and risk managers can use these diagnostics as interpretable, non-price-based risk indicators. A decline in sequential complexity (PE) may serve as an early warning of herd-like market dynamics, while an increase in distributional divergence (KL) indicates a structural break that may require recalibration of risk models dependent on recent historical data. Integrating these insights, either directly through diagnostic dashboards or indirectly through the model’s calibrated probabilistic outputs, supports more adaptive asset allocation, regime-aware hedging, and proactive risk governance.

For policymakers and regulators, the framework provides a quantitative tool for monitoring market integrity and evaluating the systemic effects of new ESG-related interventions. Because the diagnostics capture how informational diversity and structural dynamics evolve over time, they can be used to assess how policy actions, disclosure mandates, or regulatory adjustments influence market stability and efficiency. A sustained decline in SE may signal a contraction in informational diversity, reflecting lower transparency or growing consensus among market participants conditions that can precede reduced resilience or potential coordination risk. Likewise, persistently low PE can indicate synchronized trading behavior and diminished informational efficiency, warranting closer supervisory attention. In contrast, a sharp rise in KL divergence may reveal that a policy announcement or external event has materially altered the return-generating structure, signaling the onset of a regime transition. By integrating these information-theoretic diagnostics into ongoing monitoring and evaluation frameworks, policymakers can better identify early signs of stress, design more adaptive regulatory responses, and promote the long-term transparency and resilience of ESG financial systems.

### 5.4. Limitations and Future Research

Despite its robust design and compelling findings, this study has several limitations that motivate future work. First, the analysis was confined to a single emerging-market ESG index within the observed study period. While this provided an effective stress test for the types of volatility and regime shifts present in the sample (including COVID-19 and post-2022 turbulence), future research should validate the framework’s generalizability across a broader portfolio of ESG indices. This would also test the model’s robustness against systemic shocks or liquidity disruptions not observed within the current data window. Second, the design focused on three information-theoretic signals (SE, PE, KL) used as features; expanding to alternative complexity measures (e.g., Tsallis entropy, multifractal descriptors) may yield additional diagnostic or predictive value. Third, the macro-financial and technical baseline was intentionally parsimonious. This design limits a broader assessment of the model’s sensitivity to other categories of macro shocks (e.g., sudden interest rate hikes or major geopolitical events) that are only partially captured within the study period. Evaluating richer factor sets and optimized technical indicators would provide a stronger comparator and may uncover interactions with the information-theoretic layer. Fourth, the empirical strategy relied on a calibrated XGBoost classifier, which is widely regarded as the state-of-the-art approach for tabular learning [121,122]. Future research could extend this framework by embedding the proposed entropy-based signals into sequence-oriented architectures such as LSTMs, Temporal Convolutional Networks, or Transformer-based models to examine whether long-range temporal dependencies provide additional predictive value beyond the lag-structured representation adopted here. Finally, extending the targets beyond direction to return magnitude and volatility would further test decision relevance.

## 6. Conclusions

This study demonstrates that a complexity-aware, leakage-aware forecasting framework that embeds information-theoretic diagnostics can materially improve the reliability of next-day ESG index direction predictions in an emerging-market setting. By augmenting a macro-technical baseline with SE, PE, and KL divergence, the framework delivers forecasts that are not only more accurate but also demonstrably more stable and better calibrated, highlighting the value of making informational states and transitions explicit in regime-dependent markets. Empirical evidence from NCV and held-out testing supports this conceptual argument.

The augmented model exhibited superior discrimination on the strictly held-out test set, achieving a +38.5% gain in the MCC and a +12.8% improvement in BAcc. Beyond point discrimination, model reliability and calibration were both enhanced: nested cross-validation analyses showed that the performance-to-stability ratio more than doubled (>100%) across key metrics, while Brier losses decreased significantly (Δ = −0.014). The improvement was further supported by a fold-stratified McNemar’s exact test (*p* < 0.001), indicating consistently fewer misclassifications. Collectively, these findings confirm that incorporating explicit informational diagnostics strengthens not only predictive accuracy but also the temporal stability and probabilistic reliability of ESG index forecasts under varying market regimes. Taken together, these results demonstrate a generalizable and practically relevant modeling principle.

Beyond its immediate application to Türkiye’s BIST Sustainability Index, the approach suggests a portable and potentially generalizable blueprint for regime-sensitive prediction in sustainable finance, advancing methodological depth while maintaining applied relevance. Future applications may extend this framework to other national or global ESG indices and integrate it into real-world trading or risk-monitoring systems that require regime-aware and probabilistically calibrated decision support. In doing so, the proposed approach provides a scalable foundation for linking theoretical advances in information theory with the practical demands of sustainable investment and financial stability analysis. In an era when sustainable finance is rapidly gaining importance yet remains fraught with volatility and uncertainty, this framework provides a principled pathway for navigating one of modern finance’s most complex and consequential frontiers.

## Figures and Tables

**Table 1:** Summary of multi-scale determinants of ESG performance.

Determinant Category	Key Variables/Examples	Representative Literature
Macro-Financial & Market	Commodity and energy prices (oil, gold), exchange and interest rates, economic policy uncertainty, systemic shocks (e.g., pandemics, crypto spillovers).	[17,18,19,27,28,29,30,31,32,33,34]
Institutional & Structural	Economic growth, institutional quality, SDG alignment, demographic and public finance indicators, higher education, circular economy, renewable energy systems.	[35,36,37,38,39,40,41,42,43]
Corporate & Firm-Level	Financial structure (debt, liquidity), income diversification, intellectual capital efficiency, profitability, capital structure, firm performance.	[44,45,46,47,48,49,50,51]
Social, Environmental & Behavioral	Corporate social responsibility, employee engagement, social media activity, environmental disclosure, governance transparency.	[52,53,54,55]

**Table 2:** Recent ML- and DL-based frameworks for ESG forecasting.

Source	Determinant Type	Key Variables/Drivers	ESG Forecasting Models
[9]	ESG-focused Portfolios	Stock Returns, ESG Ratings, Portfolio Weights	DRIP with Multivariate Bidirectional LSTM
[15]	Fundamental, Technical, and Macroeconomic Drivers of ESG Index Volatility	Cboe Volatility Index, Interest Rate, Civilian Unemployment Rate, Consumer Sentiment Index, US Dollar Index, Technical Indicators	LSTM, GRU, CNN
[20]	ESG Newsflow–Driven Volatility Determinants	ESG-Related Financial News, Textual Features Extracted from Newsflow, Transformer-Based Language Representations	ESG2Risk Deep Learning Pipeline
[56]	Technical Indicators-Based Market Drivers	Technical Indicators	Decision Tree, Random Forest, AdaBoost, XGBoost, SVC, Naïve Bayes, KNN, Logistic Regression, ANN, RNN, LSTM
[57]	Financial Ratios & Industry-Specific Drivers	Profitability Ratios, Liquidity Ratios, Leverage Ratios, Management Efficiency Ratios, Fraud Checks, İndustry Code, Company Size (96 Financial And Industry-Related Indicators)	Decision Tree, Naïve Bayes, ANN
[58]	Urban Sustainability Indicators	Environmental, Social, and Economic Indicators	Decision Tree, ANN, SVM
[59]	Financial Sustainability of Microfinance Institutions	Operational, Financial, and Institutional Variables Of Microfinance Institutions	Random Forest, Quantile Random Forest, Linear Regression, Partial Least Squares, Stepwise Linear Regression, Elastic Net, Bayesian Ridge Regression, KNN, SVR
[60]	Financial Sustainability of Banks	Loans and Leases, Interest Income, Total Liabilities, Total Assets, Market Capitalization, Revenue To Assets, Revenue Per Share	Random Forest Classification, SHAP-based Feature Analysis, Three-Stage Network DEA
[61]	ESG Performance and Investment Decisions	ESG Variables	Light Gradient Boosting Machine, Local Outlier Factors, LSTM, GRU
[62]	Corporate ESG Disclosure and Communication	ESG-Related Sentences in Earnings Calls	Neural Language Modeling
[63]	Systemic Banking Risk & ESG Factors	ESG Risk Score, Inflation Rate, Unemployment Rate, House Prices, Current Account Balance/GDP Ratio	Interpretable Multivariate LSTM with Focal Loss

**Table 3:** Definitions and expected roles of technical indicators.

Definition	Formula
Trend-Based Technical Indicators	
EMA is a trend-following indicator that applies exponentially decaying weights to past observations [93,94]. Unlike SMA (equal weights), EMA emphasizes recent data, enhancing responsiveness while smoothing noise. It captures short- to intermediate-term directional momentum.	${EMA}_{t}=\alpha P_{t}+\;\left(1-\alpha \right){\times EMA}_{t-1}.$ where Pt denotes the closing price and α = 2/(n + 1) is the smoothing coefficient with n lookback window.
PSAR captures trend direction and potential reversals; dots below price → uptrend, above → downtrend. Also used as a trailing stop [95,96,97].	Uptrend: ${SAR}_{t}={SAR}_{t-1}+\alpha \left({EP}_{t-1}-{SAR}_{t-1}\right).\;$ Downtrend: ${SAR}_{t}={SAR}_{t-1}-\alpha \left({SAR}_{t-1}-{EP}_{t-1}\right).$ where EP is the extreme point and α is the acceleration factor.
Momentum-Based Technical Indicators	
RSI is a momentum oscillator, bounded between 0 and 100, that measures the speed and change of price movements. It is used to identify overbought (>70) and oversold (<30) conditions movements [98].	$RSI=100-\left(\frac{100}{1+RS}\right)$ where RS is the ratio of average gains to average losses over the lookback period [99].
Williams %R is bounded between 0 and −100, that measures the current closing price in relation to the high/low range over a past period n. It is used to identify overbought (>−20) and oversold (<−80) level [94].	$\%R_{t}=\left(\frac{H_{n}-P_{t}}{H_{n}-L_{n}}\right)\times \left(-100\right)$ where Pt is the closing price at time t, Hn is the highest price over the lookback period n, and Ln is the lowest price over the same period.
Volatility-Based Technical Indicators	
ATR is a measure of market volatility that incorporates price gaps. It quantifies the degree of price movement or variability, rather than the direction. High values indicate high volatility [94,100,101,102].	${TR}_{t}\;=\;\max\;\left\{\left(H_{t}-L_{t}\right),\;\mathrm{abs}\left(H_{t}-P_{t-1}\right),\;\mathrm{abs}\left(L_{t}-P_{t-1}\right)\right\}$ ${ATR}_{t}=\frac{{\left(n-1\right)ATR}_{t-1}+{TR}_{t}}{n}$ where Ht is the current high, Lt the current low, Pt-1 the previous close, TRt the true range at time t, and n the lookback period.

**Table 4:** Summary of the final engineered feature set.

Category	Features	Preprocessing Notes
Macroeconomic	exchangerate, gold, oil	Raw levels
Technical	EMA, RSI, ATR, WILLR	Optimized lookback windows
Technical indicator (fixed)	PSAR	Standard configuration
Information-theoretic	SE, PE, KL divergence	Computed on daily returns

**Table 5:** Diagnostic tests for daily ESG index return dynamics (2014–2024).

Test	Statistic/Setting	*p*-Value	Conclusion (α = 0.05)
ADF	ADF = −50.87	<0.001	Stationary; unit root rejected
Kendall-Tau	tau = 0.029	0.024	Upward trend (significant)
Ljung–Box	Lag 10	0.593	No autocorrelation (≤lag 10)
Ljung–Box	Lag 20	0.017	Serial dependence (lag 20)
Ljung–Box	Lag 50	0.022	Serial dependence (lag 50)

**Table 6:** NCV results for baseline and augmented specifications with calibrated probabilities.

Metric	Baseline Model	Augmented Model
F1 Score	0.6648 ± 0.0578	0.6646 ± 0.0193
BAcc	0.6286 ± 0.0451	0.6461 ± 0.0225
MCC	0.2744 ± 0.1010	0.2940 ± 0.0427
ROC AUC	0.6957 ± 0.0574	0.7143 ± 0.0342

**Table 7:** Fold-level robustness of calibration metrics (NCV, calibrated predictions).

Metric	HL Median Δ (Aug − Base)	90%BCa CI (HL)	Wilcoxon *p*
Brier	−0.01098	[−0.02784, −0.00610]	0.0625
ECE	−0.02797	[−0.06678, −0.01868]	0.0625

**Table 8:** Fold-level stability contrasts (ΔCV%) under NCV with calibrated probabilities.

Metric	CV% (Baseline)	CV% (Augmented)	ΔCV%	90%BCa CI	Interpretation
F1 Score	8.69	2.91	−5.78	[−8.22, −4.15]	Aug more stable
BAcc	7.18	3.48	−3.70	[−5.46, −0.73]	Aug more stable
MCC	36.81	14.51	−22.29	[−31.48, −11.04]	Aug more stable
ROC AUC	8.24	4.79	−3.45	[−5.00, −2.22]	Aug more stable

**Table 9:** Performance-to-stability ratio R under NCV with calibrated probabilities.

Metric	R (Baseline) [BCa CI]	R (Augmented) 90% [BCa CI]	% Improvement
F1 Score	11.51 [9.97, 12.76]	34.35 [27.26, 40.97]	+198.4%
BAcc	13.93 [9.49, 21.61]	28.72 [21.17, 52.03]	+106.2%
MCC	2.72 [1.88, 3.67]	6.89 [4.80, 13.27]	+153.6%
ROC AUC	12.13 [8.86, 16.27]	20.89 [13.93, 29.64]	+72.2%

**Table 10:** Prediction-level calibration contrasts (pooled out-of-sample; calibrated probabilities).

Metric	Mean Δ (Aug − Base)	90%BCa CI	Wilcoxon *p*	Perm *p*	Interpretation
Brier	−0.0140	[−0.0199, −0.0084]	0.0037	0.0001	Aug better
ECE	−0.0287	[−0.0440, −0.0117]	†	†	Aug better

Note: † ECE@10 is a distribution-level scalar that yields a single value per evaluation set; per-observation paired tests (Wilcoxon/sign-flip permutation) are therefore not applicable. Inference relies on the BCa bootstrap confidence interval for the paired difference Δ = Aug − Base.

**Table 11:** Definitive Out-of-Sample Performance.

Model	F1	BAcc	ROC-AUC	MCC
XGB-Calib (Baseline)	0.7060	0.5480	0.7210	0.2080
XGB-Calib (Augmented)	0.7190	0.6180	0.7230	0.2880
Δ% (Aug − Base)	(+1.8%)	(+12.8%)	(+0.3%)	(+38.5%)

## Data Availability

The original contributions presented in this study are included in the article. Further inquiries can be directed to the corresponding authors.

## Supplementary Materials

The following supporting information can be downloaded at: https://www.mdpi.com/article/10.3390/e27111164/s1, Table S1. Hyperparameter search space for the Time-Respecting Calibrated XGBoost model; Table S2. Final Hyperparameters; Table S3. NCV results for baseline and augmented specifications with calibrated probabilities (7-day and 21-day entropy windows); Table S4. Summary of fold- and prediction-level robustness analyses (7-day entropy window); Table S5. Summary of fold- and prediction-level robustness analyses (21-day entropy window).

## Appendix A. Information-Theoretic Feature Definitions

The formal definitions of Shannon entropy (SE), permutation entropy (PE), and Kullback–Leibler (KL) divergence used in this study are given below:

Shannon Entropy (SE):

$$H_{SE}=\;-\sum_{i=1}^{K}p_{i}\ln\left(p_{i}\right)$$

Permutation Entropy (PE):

$$H_{PE}=\;-\frac{1}{\log_{2}d!}\sum_{j=1}^{d!}p\left(\pi_{j}\right)\log_{2}\left(p\left(\pi_{j}\right)\right)$$

Kullback–Leibler Divergence (KL):

$$D_{KL}\left(P∥Q\right)=\sum_{i=1}^{K}p_{i}\ln\left(\frac{p_{i}}{q_{i}}\right)$$

Parameters used in this study:

- Number of bins K=10;
- Numerical offset $\varepsilon =10^{-12}$ (for stability);
- Embedding dimension d=3;

Time lag τ=1.

## Appendix B. Time-Respecting Calibration Protocol

Let the development span (training+validation) contain indices $t=1,\ldots ,T_{dev}$, and let $\alpha \in \left(0,1\right)$ denotes the calibration fraction of this span (α). The cut-off point is defined as $t_{c}=\left\lfloor \left(1-\alpha \right)T_{dev}\right\rfloor$. The calibration protocol proceeded as:

1. Fit the base XGBoost f ${\left\{\left(X_{t},y_{t}\;\right)\right\}}_{t=1}^{t_{c}}$.
2. Score the later, disjoint calibration holdout ${\left\{\left(X_{t},y_{t}\;\right)\right\}}_{t=t_{c}+1}^{T_{dev}}:s_{t}=f\left(X_{t}\right)$ (uncalibrated margins/probabilities as implemented).
3. Fit a mapping g on ${\left\{\left(s_{t},y_{t}\;\right)\right\}}_{t=t_{c}+1}^{T_{dev}}$ and apply it forward, yielding calibrated probabilities ${\hat{p}}_{t}=g\left(f\left(X_{t}\right)\right)$.

## Appendix C. Evaluation Metrics

Directional-accuracy metrics:

$$BAcc=\frac{1}{2}\left(\frac{TP}{TP+FN}+\frac{TN}{TN+FP}\right)$$

$$Precision=\frac{TP}{TP+FP}$$

$$Recall=\frac{TP}{TP+FN}$$

$$F1=\frac{2\cdot Precision\cdot Recall}{Precision+Recall}$$

$$MCC=\frac{TP\cdot TN-FP\cdot FN}{\sqrt{\left(TP+FP)(TP+FN)(TN+FP)(TN+FN\right)}}$$

Discrimination metric:

$$AUC=\frac{1}{n_{1}n_{0}}\sum_{i:y_{i}=1}\sum_{j:y_{j}=0}(1\left\{{\hat{s}}_{i}>{\hat{s}}_{j}\right\}+\frac{1}{2}\;1\left\{{\hat{s}}_{i}={\hat{s}}_{j}\right\}$$
