# Supplementary material for: Information-Theoretic ESG Index Direction Forecasting: A Complexity-Aware Framework
Source: Entropy (Basel). 2025 Nov 17;27(11):1164. doi: 10.3390/e27111164 (PMC12651845; doi:10.3390/e27111164)
Supplement: Supplementary file 1 [file entropy-27-01164-s001.zip › entropy-3935620-supplementary.pdf]

Supplementary Materials:

Table S1. Hyperparameter search space for the Time-Respecting Calibrated XGBoost model.

Component	Hyperparameter	Distribution / Type	Range / Support	Notes
**Base learner (XGBoost)**	n_estimators	randint	300–1500 (integers)	—
	max_depth	randint	2–6 (integers)	—
	min_child_weight	randint	1–7 (integers)	—
	gamma	loguniform	1e−4–3.0	Minimum loss reduction to split
	learning_rate	loguniform	0.005–0.20	Eta
	subsample	uniform	0.60–1.00	Row subsampling
	colsample_bytree	uniform	0.60–1.00	Feature subsampling per tree
	reg_lambda	loguniform	0.001–10	L2 regularization
	reg_alpha	loguniform	0.0001–1.0	L1 regularization
	max_bin	categorical	{128, 256, 512}	Histogram bin count
	tree_method	fixed	hist	Histogram-based growth
	scale_pos_weight	computed	neg/pos	Handles class imbalance
**Calibration layer**	calib_method	categorical	{isotonic, platt}	PAVA vs. logistic (Platt)
	calib_frac	uniform	0.15–0.30	Past-only terminal calibration slice
	min_calib	randint	100–200 (integers)	Minimum calibration sample size
**Resampling & search**	inner CV	fixed	TimeSeriesSplit (n_splits = 3)	Chronological, no shuffling
	outer CV	fixed	TimeSeriesSplit (n_splits = 5)	Performance estimation folds
	search budget	fixed	n_iter = 200	200 random draws per inner loop

Table S2. Final Hyperparameters.

Parameter	Baseline (no-entropy)	Augmented (entropy)
n_estimators	1106	768
max_depth	2	5
min_child_weight	2	6
gamma	0.0004	0.0045
learning_rate	0.0145	0.0090
subsample	0.6135	0.7073
colsample_bytree	0.9302	0.8870
reg_lambda	0.0258	0.1456
reg_alpha	0.0029	0.1193
max_bin	256	256
calib_method	isotonic	platt
calib_frac	0.1617	0.1602
min_calib	198	125

Table S3. NCV results for baseline and augmented specifications with calibrated probabilities (7-day and 21-day entropy windows).

Window	Metric	Baseline Model	Augmented Model
7-Day	F1 Score	0.6461 ± 0.0528	0.6828 ± 0.0442
21-Day		0.6977 ± 0.0391	0.6919 ± 0.0458
7-Day	BAcc	0.6313 ± 0.0406	0.6304 ± 0.0333
21-Day		0.6246 ± 0.0576	0.6377 ± 0.0255
7-Day	MCC	0.2765 ± 0.0835	0.2858 ± 0.0536
21-Day		0.2851 ± 0.0847	0.2898 ± 0.0396
7-Day	ROC AUC	0.7030 ± 0.0499	0.7282 ± 0.0297
21-Day		0.7202 ± 0.0361	0.7359 ± 0.0296

Table S4. Summary of fold- and prediction-level robustness analyses (7-day entropy window).

Category	Metric / Test	Statistic	Interpretation
Fold-Level Robustness of Calibration Metrics	Δ Brier (Aug–Base)	HL = −0.007737[−0.02696, −0.01622];*p* = 0.0625	Augmented better
	Δ ECE (Aug–Base)	HL = −0.0075 [−0.0473, −0.0145];*p* = 0.4375	Small Aug advantage
Fold-Level Stability	ΔCV % (BAcc)	Δ CV % = −1.16 [−1.78, −0.53]; Perm p = 0.1257	Augmented more stable
Performance-to-Stability Ratio	R (BAcc Aug vs Base)	R(Base) = 15.54; R(Aug) = 18.95 (+21.9 % *Augmented advantage*)	Higher efficiency of performance
Prediction-Level Calibration Contrasts	Δ Brier (Aug–Base)	Mean Δ = −0.02146 [−0.02696, −0.01622]; *p* < 0.01	Augmented better
	Δ ECE (Aug–Base)	Mean Δ = −0.0297[−0.0473, −0.0145]	Augmented better

Table S5. Summary of fold- and prediction-level robustness analyses (21-day entropy window).

Category	Metric / Test	Statistic	Interpretation
Fold-Level Robustness of Calibration Metrics	Δ Brier (Aug–Base)	HL = −0.00556 [−0.00890, −0.00213];p = 0.0625	Augmented better
	Δ ECE (Aug–Base)	HL = −0.09253 [−0.30915, −0.10636]; p = 0.0610	Augmented better
Fold-Level Stability	ΔCV % (BAcc)	ΔCV % = −5.22 [−7.22, −1.75]; Perm p = 0.0635	Augmented more stable
Performance-to-Stability Ratio	R (BAcc Aug vs Base)	R(Base)=10.85; R(Aug)=25.05 (+130.8 % Aug advantage)	Higher efficiency of performance
Prediction-Level Calibration Contrasts	Δ Brier (Aug–Base)	Mean Δ = −0.00537 [−0.00890, −0.00213]; Perm p = 0.0092	Augmented better
	Δ ECE (Aug–Base)	Mean Δ = −0.0056[−0.0253, 0.0046]	Small Aug advantage
